# La tumeur de Buschke-Lowenstein anorectale: à propos de 16 cas et revue de la littérature

**DOI:** 10.11604/pamj.2013.16.131.2864

**Published:** 2013-12-08

**Authors:** Noureddine Njoumi, Mohamed Tarchouli, Moulay Brahim Ratbi, Mohamed Reda Elochi, Rajae Yamoul, Hafid Hachi, Abdesslam Bougtab

**Affiliations:** 1 Service de chirurgie II, Institut National d'Oncologie, CHU Ibn Sina, Faculté de Medecine et de Pharmacie de Rabat, Maroc; 2 Service d'anatomie pathologique, HMIMV, Rabat, Maroc

**Keywords:** Tumeur de Buschke-Lowenstein, carcinomes verruqueux, HPV, Buschke-Lowenstein tumor, verrucous carcinoma, HPV

## Abstract

La tumeur de Buschke-Lowenstein est une affection rare appartenant au groupe des carcinomes verruqueux. Elle survient le plus souvent chez des sujets pubères en pleine activité sexuelle. Une infection par human papillomavirus (HPV) 6 et 11 est volontiers associée à ces tumeurs. Elle se caractérise par la fréquence des récidives et le risque de transformation maligne. Son traitement est difficile même si l'histologie confirme la bénignité. A partir de 16 observations de TBL et d'une revue de la littérature, les auteurs soulignent les aspects épidémiologiques, cliniques, thérapeutiques et évolutifs de cette affection.

## Introduction

La tumeur de Buschke-Löwenstein(TBL) ou condylome acuminé géant(CAG) est une prolifération pseudo-épithéliomateuse appartenant au groupe des carcinomes verruqueux. Sa première description remonte à 1896. C'est en 1925 que Buschke et Löwenstein en ont fait une entité caractérisée [1]. Elle est d'origine virale (HPV), de transmission sexuelle atteignant surtout les zones ano-génitales [2]. Elle se distingue des condylomes acuminés par sa prolifération plus marquée et une pénétration profonde dans les tissus sous-jacents qui peuvent alors être refoulés, et d'un carcinome épidermoïde par l'absence d'invasion histologique et de métastase [3]. Elle est caractérisée par son potentiel dégénératif et son caractère récidivant après traitement [4, 5]. Nous rapportons à ce propos notre expérience à travers une série de 16 observations, en passant en revue les données de la littérature.

## Méthodes

Cette étude porte sur 16 patients ayant été traités pour TBL à localisation ano-rectale entre le 1er janvier 2001 et le 31 décembre 2010. Nous avons étudié plusieurs paramètres dont l’âge, le sexe, le statut familial et les habitudes toxiques. Du point de vue clinique, nous avons pris en considération l'aspect de la tumeur, sa localisation, sa taille et les signes fonctionnels. L'histologie était nécessaire pour le diagnostic. Les moyens thérapeutiques ont été élucidés ainsi que l’évolution après ces différents traitements.

## Résultats

L’âge moyen de la population étudiée est de 44 ans (extrêmes: 24-70), la moitié des malades se situe dans la tranche d’âge comprise entre 40 et 50 ans. Ils sont répartis en 15 hommes et une femme. Tous des patients sont mariés et aucun d'entre eux n'a avoué une pratique homosexuelle. Le tabagisme est presque constant et l'alcoolisme est signalé dans trois cas.

**Figure 1:**
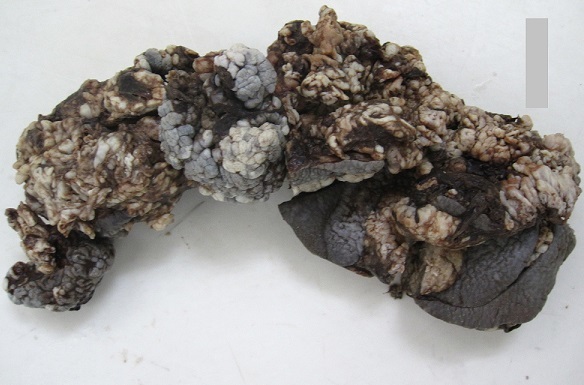
Aspect macroscopique après fixation de la tumeur

La lésion reproduit dans toutes nos observations l'aspect végétant, bourgeonnant en crêtes de coq ou en choux-fleurs (Figure 1). Elle est infectée dans 12.5% et hémorragique dans 18% des cas. La taille de la tumeur varie entre 5 et 25 cm, elle est supérieure à 10 cm dans 64% des cas. La localisation au niveau de la marge anale est constante. Dans deux cas (12.5%) la tumeur fuse en haut et atteint l'ampoule rectale. Les organes génitaux externes sont envahis par le processus tumoral chez quatre patients (25%).

Toutes les lésions ont été diagnostiquées comme condylome acuminé géant. La biopsie préopératoire a confirmé ce diagnostic dans tous les cas, et a montré une dégénérescence en un carcinome épidermoïde dans deux cas. L’étude histologique de la pièce opératoire a montré un carcinome epidermoide chez deux patients dont la biopsie antérieure était en faveur d'un CAG. La récidive a dégénéré dans un seul cas. Dans un autre cas la TBL était associée à un adénocarcinome rectal.

Quatre-vingt-sept pour cent des patients (14 malades) ont bénéficiés d'un traitement chirurgical. Il a consisté chez 13 patients en une exérèse large. Le champ d'exérèse était protégé par une colostomie dans deux cas. La résection était palliative chez un patient présentant une énorme TBL envahissant le système sphinctérien et fusant vers le rectum. Dans le cas ou la TBL était associée à un adénocarcinome rectale, une amputation abdomino-périnéale élargie à la paroi postérieure du vagin a été réalisée, complétée par une radiochimiothérapie.

L’électrocoagulation a été indiquée pour des lésions condylomateuses loin du champ d'exérèse dans 12,5% des cas. La radiothérapie était exclusive dans deux cas de TBL dégénérée, et adjuvante dans deux autres cas qui ont présenté un condylome géant banal à la biopsie, alors que l’étude histologique de la pièce opératoire a montré une transformation maligne de la TBL.

Le suivi a été possible chez 13 patients, trois de nos malades ont été perdus de vue. Nous n'avons relevé aucun cas de décès postopératoire. L’évolution était marquée par l'apparition d'une sténose anale dans trois cas. Sur les 13 patients dont nous avons pu suivre l’évolution, 46%( 06 cas) ont bien évolué avec un recul de 98 mois (extrêmes 20- 128). 38,4% (05cas) ont présenté des récidives dans un délai moyen de 18 mois (extrêmes 10-34), l’étude anatomopathologique de ces récidives a montré un aspect compatible avec une TBL chez 04 patients, trois d'entre eux ont subit une exérèse chirurgicale alors que le quatrième a été perdu de vue. La récidive a dégénéré dans un seul cas avec évolution ganglionnaire, le traitement était palliatif à base d'une radiothérapie.

La régression a été incomplète pour les deux patients traitée par radiothérapie exclusive.

## Discussion

La TBL est une affection relativement rare et toujours précédée de condylomes acuminés. Son incidence annuelle semble être de 0,1% parmi la population adulte active sexuellement [5, 6]. Elle survient à tout âge après la puberté et prédomine entre les 4e et 6e décennies [7]. L’âge moyen des malades de notre série est de 44 ans, ce qui reflète le jeune âge des patients en plein activité sexuelle.

L'infection peut atteindre les deux sexes, elle se voit fréquemment chez le sexe masculin [8]. Dans notre étude nous rapportons un cas féminin sur 16 patients. Le développement, la persistance et les récidives des condylomes dépondent largement du statut immunitaire de l'hôte. L'immunodépression, l'inflammation chronique, le manque d'hygiène et l'infection à VIH semblent être des facteurs de risque de cette affection [4, 8–11]. L'implication du papillomavirus et en particulier de ses sérotypes l'HPV 6 et 11 est admise dans la genèse de la TBL, Le potentiel oncogène de ces deux virus est faible contrairement à celui de l'HPV 16 et HPV 18 [3, 11, 12]. L’étude virologique n'a pas été faite dans notre série.

La TBL se localise le plus souvent au niveau des organes génitaux externes et principalement au niveau de la verge. La localisation anorectale reste moins fréquente mais elle n'est pas rare. Cette localisation est retrouvée dans les deux sexes avec une prédominance masculine [3, 5, 13] comme c’était le cas dans notre série. Chez l'homme, la TBL se localise dans 81 à 94% des cas au pénis et dans 10 à 17% des cas à la région ano-rectale. Chez la femme, la localisation est essentiellement vulvaire dans 90% des cas contrairement à la localisation ano-rectale qui reste moins fréquente [6]. Dans notre série la localisation au niveau de la marge anale est constante avec deux cas d'atteinte rectale et quatre cas d'envahissement des organes génitaux externes.

Le patient consulte parfois pour douleur péri-anale, prurit, rectorragie, écoulement purulent, perte de poids et/ou la palpation d'une masse périnéale [14]. La tumeur est toujours précédée de lésions condylomateuses grisâtres ou rosées, évoluant progressivement pour prendre un aspect papillomateux, irrégulier, en chou-fleur [3, 5]. Elle évolue en surface et en profondeur, ce qui marque sa différence des condylomes acuminés banaux. L'extension peut se faire vers le scrotum ou la vulve, le sillon interfessier, les fesses, voire le rectum et le pelvis. En surface elle peut donner naissance à une énorme tumeur d'une dizaine de centimètres. En profondeur la tumeur évolue en détruisant et refoulant les structures avoisinantes sans les infiltrer [3, 5, 8, 13].

En fonction de localisation, le bilan d'extension peut comporter outre la palpation des aires ganglionnaires, une rectoscopie, un examen gynécologique, une tomodensitométrie pelvienne ou une résonance magnétique nucléaire [5].

**Figure 2:**
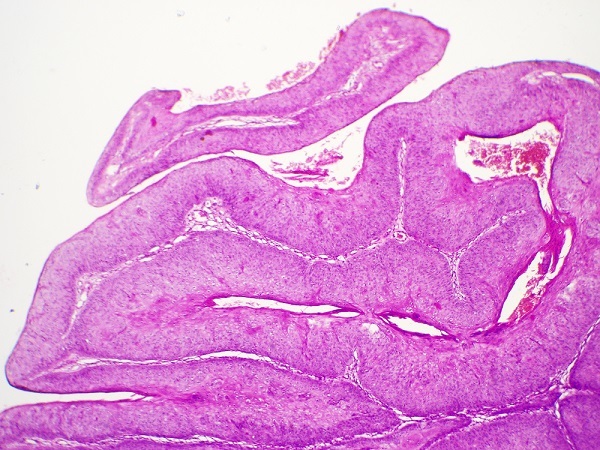
Epiderme papillomateux acanthosique (G×20)

**Figure 3:**
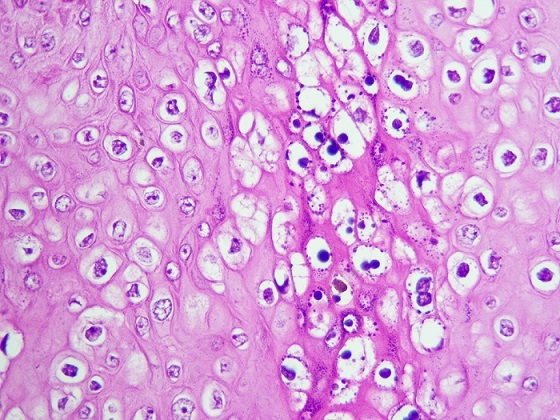
Présence de nombreuses koïlocytes (G×40)

Sur le plan histologique, c'est une tumeur malpighienne parfaitement limitée, caractérisée par une hyperplasie épithéliale considérable parfois pseudo-épithéliomateuse, une hyperacanthose (Figure 2), une hyperpapillomatose et des koïlocytes qui sont des marqueurs pathognomoniques de l'infection par HPV (Figure 3), cependant leur présence n'est pas constante. La membrane basale reste intacte ce qui preuve la bénignité de la tumeur malgré son comportement malin [6, 13, 14].

L’évolution est lente, elle peut être grevée de plusieurs complications dont la dermite, l'infection, la fistulisation aux organes de voisinage, la nécrose, la sténose anale et l'hémorragie [7, 13]. La transformation maligne constitue l'un des risques évolutifs [11]. Elle a été rapportée dans 30% à 56% des cas [7, 9, 11, 13, 14]. Cette incidence est de 31,2% dans notre série.

La tumeur de Buschke- Lowenstein pose le problème de diagnostic différentiel avec d'autres pathologies [7]. En effet, certaines lésions tumorales (les épithéliomas spino-cellulaires) ou infectieuses (la syphilis dans sa forme secondaire, la tuberculose verruqueuse et végétante, la maladie de Nicolas Favre, la donovanose ou granulome inguinal, l'amibiase ano-génitale) peuvent simuler à l’étape clinique la TBL [6]. La recherche d'autres infections sexuellement transmissibles est systématique (HIV, chlamydia trachomatis, syphilis) [6, 8]. Dans notre série, aucun cas d'association à l'HIV n'a été rapporté.

Le traitement des TBL est souvent difficile, même si l'histologie confirme la bénignité [4]. La chirurgie reste le traitement de choix pour la majorité des auteurs [3, 7, 9]. Elle doit être suffisamment large voire mutilante pour écarter le spectre de récidive et espérer une guérison définitive. Elle est variable selon la localisation [7, 8]. Dans les localisations péri-anales, une exérèse avec conservation du sphincter et reconstruction est réalisée aussi souvent que possible, mais des interventions plus lourdes à type d'amputation du rectum ou d'amputations abdominopérinéales sont parfois nécessaires [3]. Le caractère complet de l'exérèse chirurgicale de la TBL doit être confirmé par l'examen anatomopathologique de la pièce opératoire. Si l'exérèse chirurgicale est incomplète, la reprise chirurgicale est indiquée [8]. Les patients présentant des lésions étendues avec plusieurs trajets fistuleux et/ou surinfection peuvent nécessiter une colostomie temporaire de décharge [9].

Les topiques locaux (podophyline, 5FU), l’électrocoagulation, la cryothérapie, et la destruction au laser largement utilisés dans le traitement des condylomes banaux sont inefficaces dans le traitement de la TBL [3, 4, 7, 6, 15]. Ces moyens ont l'inconvénient majeur de ne pas fournir un tissu pour l'analyse histologique [8, 15].

La chimiothérapie à base de méthotrexate ou de bléomycine peut être utilisée mais elle reste sans véritable apport, elle est surtout indiquée en préopératoire afin de réduire le volume tumoral et de diminuer l'agressivité de l'acte chirurgical [6]. L'utilisation de la radiothérapie est controversée [3, 4, 8]. Elle est utilisée en préopératoire pour diminuer la masse tumorale, ou en dernier recours pour des tumeurs non opérables [3, 15]. Pour certains auteurs, L'association de la radiothérapie à la chimiothérapie en neoadjuvant pourrait donner des résultats remarquables, elle a été utilisée avec succès pour traiter des TBL dégénérées [9, 13]. Pour d'autres, La chimiothérapie et la radiothérapie ne doivent être administré que dans les TBL non résécables ou récidivantes puisque leur efficacité n′a pas été pleinement documenté [16].

L'immunothérapie par autovaccination semble avoir une certaine efficacité dans le traitement des condylomes anciens et récidivants [6].

Les récidives sont l'une des caractéristiques de cette tumeur, elles sont la conséquence directe d'un geste chirurgical trop limité [8]. Le risque de récidives après excision est de 60 à 66% [9, 11], ce taux est de 38,4% dans notre série.

## Conclusion

La TBL est une prolifération épithéliale condylomateuse d'origine virale dont le génie évolutif est incertain avec un risque de transformation en carcinome épidermoide. Sa prévention est impérative basée sur le traitement des condylomes acuminés et la lutte contre les maladies sexuellement transmissibles. Le traitement doit être précoce, il est essentiellement chirurgical nécessitant une large exérèse. Une surveillance postopératoire clinique et histologique étroite et prolongée s'impose, sans perdre de vue la notion de dégénérescence de la récidive.
